# Artificial Intelligence in Facial Palsy Treatment: A Systematic Review and Recommendations

**DOI:** 10.1097/PRS.0000000000012105

**Published:** 2025-03-18

**Authors:** Seraina L. C. Müller, Pablo Pfister, Nadia Menzi, Laurent Muller, Holger J. Klein, Riccardo Schweizer, Z-Hye Lee, Branislav Kollar, Steffen U. Eisenhardt, Dirk J. Schaefer, Tarek Ismail

**Affiliations:** Basel, Aarau, and Lucerne, Switzerland; Houston, TX; and Freiburg, Germany; From the Departments of 1 Plastic, Reconstructive, Aesthetic, and Hand Surgery; 2 Otorhinolaryngology, Head and Neck Surgery, University Hospital Basel; 3 Department of Plastic and Hand Surgery, Cantonal Hospital Aarau; 4 Department of Plastic Surgery and Hand Surgery, Cantonal Hospital Lucerne; 5 Department of Plastic Surgery, University of Texas MD Anderson Cancer Center; 6 Department of Plastic and Hand Surgery, University of Freiburg Medical Center, Medical Faculty of the University of Freiburg.

## Abstract

**Background:**

Artificial intelligence (AI) is rapidly advancing and increasingly applied in facial palsy research. However, there is no comprehensive review to guide surgeons on AI-based facial assessment tools. Although photographic standards exist, videographic standards for emotions have not been proposed. Implementing these standards is essential for improving information exchange and data comparison with the new AI tools.

**Methods:**

The authors conducted a systematic review following the Preferred Reporting Items for Systematic Reviews and Meta-Analyses guidelines, by analyzing databases including MEDLINE, Embase, and the Cochrane Central Register of Controlled Trials. The authors’ focus was on the use of AI-based facial assessment tools in patients with facial palsy who subsequently received intervention or surgery for their facial palsy. Data were evaluated descriptively, and recommendations, including videographic standards, were developed in collaboration with experts from multiple centers.

**Results:**

The authors identified 3222 articles, 35 of which met the inclusion criteria. Five AI applications analyzed static, dynamic, and chemodenervation procedures in unilateral or bilateral facial palsy. These focused on specific facial landmarks or emotion recognition from photographs and videos, but varied in the expressions and emotions analyzed. Five studies provided validation data with either healthy subjects or other outcome measurements. The authors recommend a minimum videographic assessment including the validated emotions neutral and happy.

**Conclusions:**

AI-related publications on facial palsy have significantly increased, but no consensus exists on the optimal AI-assessment software. The proposed flowchart from the authors’ systematic review can guide clinicians in decision-making. The authors recommend using the proposed videographic emotions to improve study consistency and comparability, and also encouraging further validation studies.

Since the first documented nerve transfer procedure in the late nineteenth century, surgeons have continued to introduce new techniques to improve facial function.^1^ Facial reanimation surgery includes various operative and interventional procedures in individuals with facial paralysis.^2^

Techniques to restore facial nerve function include direct nerve repair, nerve reconstruction with interposition grafts, and nerve transfers to reinnervate the original facial muscles.^3,4^ In cases of long-term paralysis (>2 years of denervation) or congenital absence of facial muscles and/or nerve, free functional muscle transplantation becomes necessary.^5^ The entire neuromuscular unit needs to be replaced, and reinnervation is achieved using several types of donor nerves such as the contralateral facial nerve, ipsilateral cranial nerves not initially associated with facial expression, or a combination. These allow for single, dual, or triple innervation strategies.^6–8^ In addition, static techniques can be applied to achieve a degree of facial symmetry.

To evaluate the efficacy of the various interventional and operative techniques, various assessment tools are widely accepted. Most current evaluation strategies include patient-reported outcome measures—such as the Facial Clinimetric Evaluation,^9^ Facial Disability Index,^10^ FACE-Q,^11^ and Synkinesis Assessment Questionnaire.^12^ Subjective physician-led scoring systems include the House-Brackmann Grading scale,^13^ Sunnybrook Facial Grading Scale,^14^ Sydney Facial Grading System,^15^ in addition to the newer eFACE, an app-based tool to grade facial paralysis based on standardized photographs.^16^ Although all of these assessment tools have been validated in the literature, all of them lack objectivity in assessing facial symmetry, function, and emotional expression. Over the past decade, advances in artificial intelligence (AI) have rendered manual marking obsolete, enabling standardized and objective analysis.^17^

Recent studies have shown promising results in automatically identifying facial landmarks and recognizing emotions in facial palsy patients—thus, marking a shift toward more objective assessments.^18–20^ Although these technological advancements are gradually being integrated into research and clinical practice, the variability in software options and the lack of clarity regarding their respective merits and drawbacks present challenges in widespread implementation. Furthermore, no videographic standard for AI assessment currently exists.

This systematic review evaluates the role of AI facial assessment tools to evaluate therapeutic interventions in facial paralysis patients. The goal is to highlight the advantages and limitations of these tools and to provide practical guidelines, including a proposed video standard for researchers to implement in clinical studies.

## PATIENTS AND METHODS

### Literature Search Strategy

This study was conducted in accordance with the Declaration of Helsinki. No institutional review board approval was required. We followed the Preferred Reporting Items for Systematic Reviews and Meta-Analyses guidelines for systematic reviews. A systematic search of MEDLINE, Embase, and the Cochrane Central Register of Controlled Trials using keywords and database-specific subject headings (last search date: February 18, 2024) was conducted. The full search strategy is listed in Supplemental Digital Content 1. (**See Figure, Supplemental Digital Content 1**, which shows the detailed search strategy for the systematic review, including database queries and the study selection process, https://links.lww.com/PRS/H922.)

### Eligibility Criteria

The systematic review includes studies that involved human patients diagnosed with facial palsy undergoing surgery or intervention (eg, botulinum toxin, transient nerve block) and examined by an automated AI-based facial assessment approach. AI in this context refers to machine learning-based systems trained on large data sets. Studies were excluded if they primarily relied on subjective grading scales or used nonvisual methods such as electroneurography or electromyography for assessment. In addition, research where the AI software was not mentioned or referenced, and editorials, letters, narrative reviews, case reports, conference abstracts, and trial registry entries, were not considered.

### Outcomes

Different AI-based facial assessment tools in facial reanimation surgery were compared, and recommendations were developed.

### Study Identification and Data Extraction

**Fig. 1.**
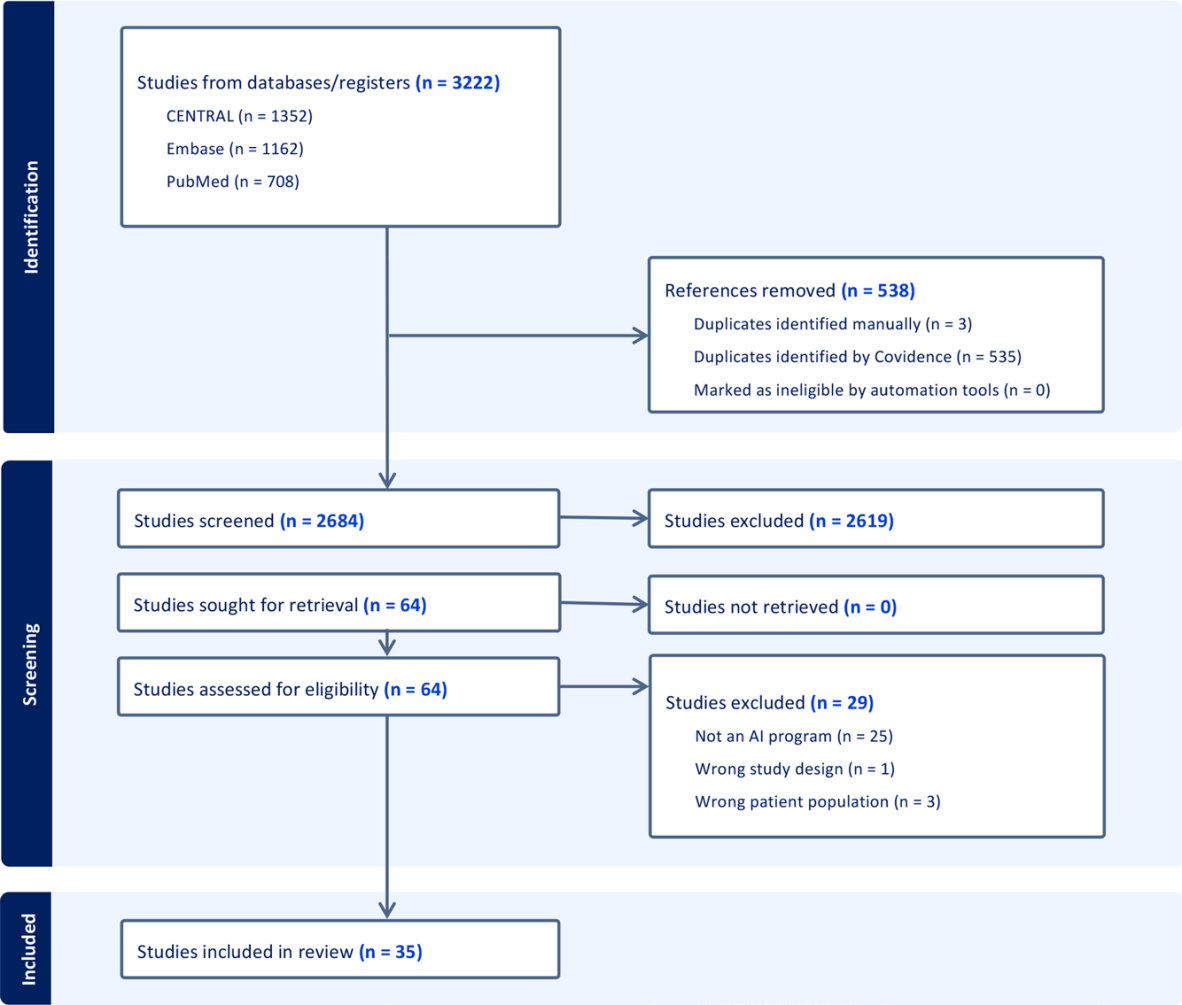
2020 Preferred Reporting Items for Systematic Reviews and Meta-Analyses flow diagram tailored for new systematic reviews. This includes the comprehensive search strategy covering databases, registers, and various sources, highlighting the use of Cochrane CENTRAL, the Cochrane Central Register of Controlled Trials.

The collected references were imported into Endnote 20 (Clarivate Analytics, 2020). The process of selecting studies for inclusion continued on Covidence, a Web-based tool for systematic reviews (Veritas Health Innovation, 2023; available at www.covidence.org, last accessed February 18, 2024). Initially, 1 reviewer independently examined all gathered references. Subsequently, 2 reviewers conducted a screening of the full-text articles identified through the initial review to assess their suitability for inclusion. Any differences in opinion regarding the inclusion of studies were resolved through discussion or the intervention of a third reviewer. The quality and risk of bias of the studies was assessed with the Risk of Bias in Non-Randomized Studies—of Intervention tool by 2 reviewers.^21^ The process adhered to the guidelines provided by the Preferred Reporting Items for Systematic Reviews and Meta-Analyses, with the selection procedure detailed in a flowchart depicted in Figure 1. For the studies deemed eligible, data extraction was carried out independently by 2 reviewers using a specifically designed spreadsheet to facilitate comparison and ensure accuracy. Any discrepancies encountered during this phase were settled through consensus or the input of a third reviewer. Recommendations and discussions were formulated in collaboration with an interdisciplinary, multicenter expert panel.

## RESULTS

### Study Characteristics

Our systematic review yielded 3222 articles from the initial database query. Following our selection process, which included a full-text screening of 64 articles, we ultimately incorporated 35 studies in this review. (**See Table, Supplemental Digital Content 2**, which shows a complete list of the 35 studies included in the systematic review, highlighting their key characteristics and relevance to the analysis, https://links.lww.com/PRS/H923.)

**Fig. 2.**
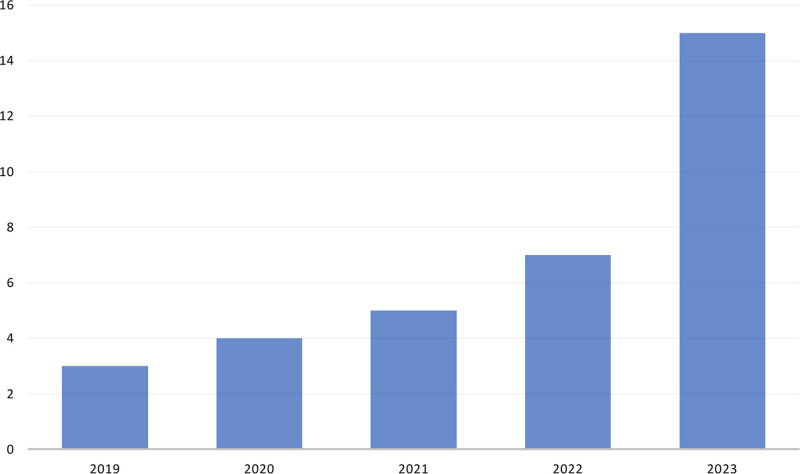
Number of studies published annually on AI-based facial assessment in facial reanimation surgery from 2019 to 2023.

The analysis showed a growing engagement with AI-based facial assessment in the surgical setting (Fig. 2). The interest in using these tools in clinical research demonstrated a rising trajectory, beginning in 2019^20,22^ with the introduction of Emotrics and Affdex.^23^ Emotrics is a software that allows analysis of symmetry and Affdex is an AI-based emotion recognition application. Furthermore, Boonipat et al. introduced FaceReader, which is also automatic emotion recognition software, for facial reanimation surgery in 2020.^17^

All of the studies had a retrospective design. In terms of quality, 1 study demonstrated a low risk of bias according to the Risk Of Bias In Non-Randomized Studies—of Intervention tool, whereas the others were assessed as having a moderate risk. Six authors conducted validation studies (**see Table, Supplemental Digital Content 2**, httsp://links.lww.com/PRS/H923).

A combined patient pool from the reviewed studies consisted of 1297 individuals who underwent facial reanimation operations or interventions and were subsequently assessed using AI technologies. The mean age was 44.2 ± 11.6 years (range, 3 to 90 years), with a gender distribution of 793 (62%) women and 477 (38%) men (data on 1 study is missing); 20 of 57 studies (57%) explicitly focused on unilateral facial palsy, whereas 8 of 35 (23%) addressed both unilateral and bilateral cases.

### Surgical Interventions

**Fig. 3.**
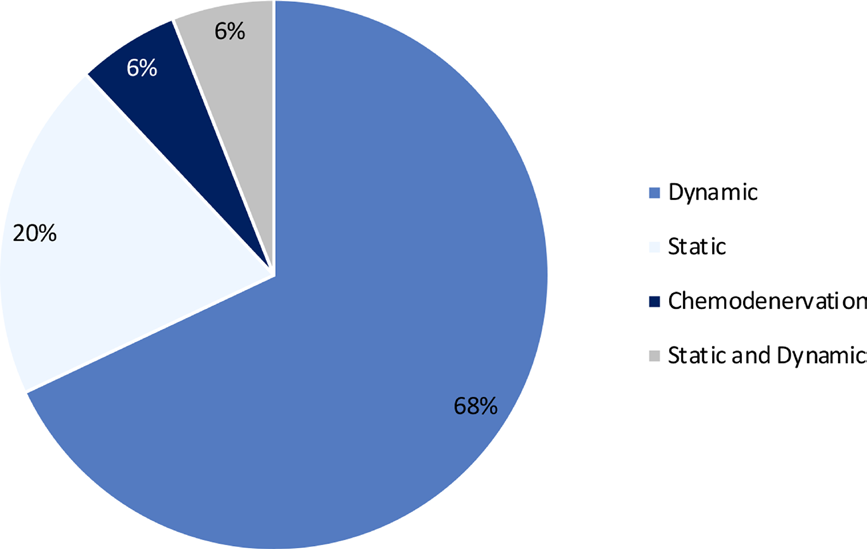
Percentage of interventions and/or operations performed in the identified studies.

The interventions consisted of dynamic facial reanimation procedures in 68% of studies (24 of 35), static procedures in 20% (7 of 35), and chemodenervation procedures in 6% of studies (2 of 35). Two studies assessed a combination of static and dynamic procedures (6% [2 of 35]) (Fig. 3). Functional muscle transfer was performed in 17 of 35 studies (49%) studies, predominantly using the free functional gracilis muscle (94% [16 of 17]). Muscles were innervated in 15 of 17 studies (88%) with cross-facial nerve grafts and in 2 of 17 studies (12%) with the masseter nerve as donor nerve. Innervation strategies varied from single to dual innervation. Nerve transfers to the facial nerve using cross-facial nerve grafts were evaluated in 1 study. Six studies reported neurectomy for static procedures. Other static approaches included lateral tarsal strip, tensor fascia lata sling, eyelid weights, and depressor anguli oris resection. Only 2 studies evaluated chemodenervation procedures: 1 involving injections of botulinum toxin, and the other using a short-lasting muscle block. The average follow-up duration for the dynamic surgery group was reported to be 30 months, in contrast to 20 months for the static surgery group. In the case of the 2 studies involving chemodenervation, the follow-up period after botulinum toxin injection was at least 20 days, whereas for muscle block, it was 45 minutes.

### AI Software Assessment

**Fig. 4.**
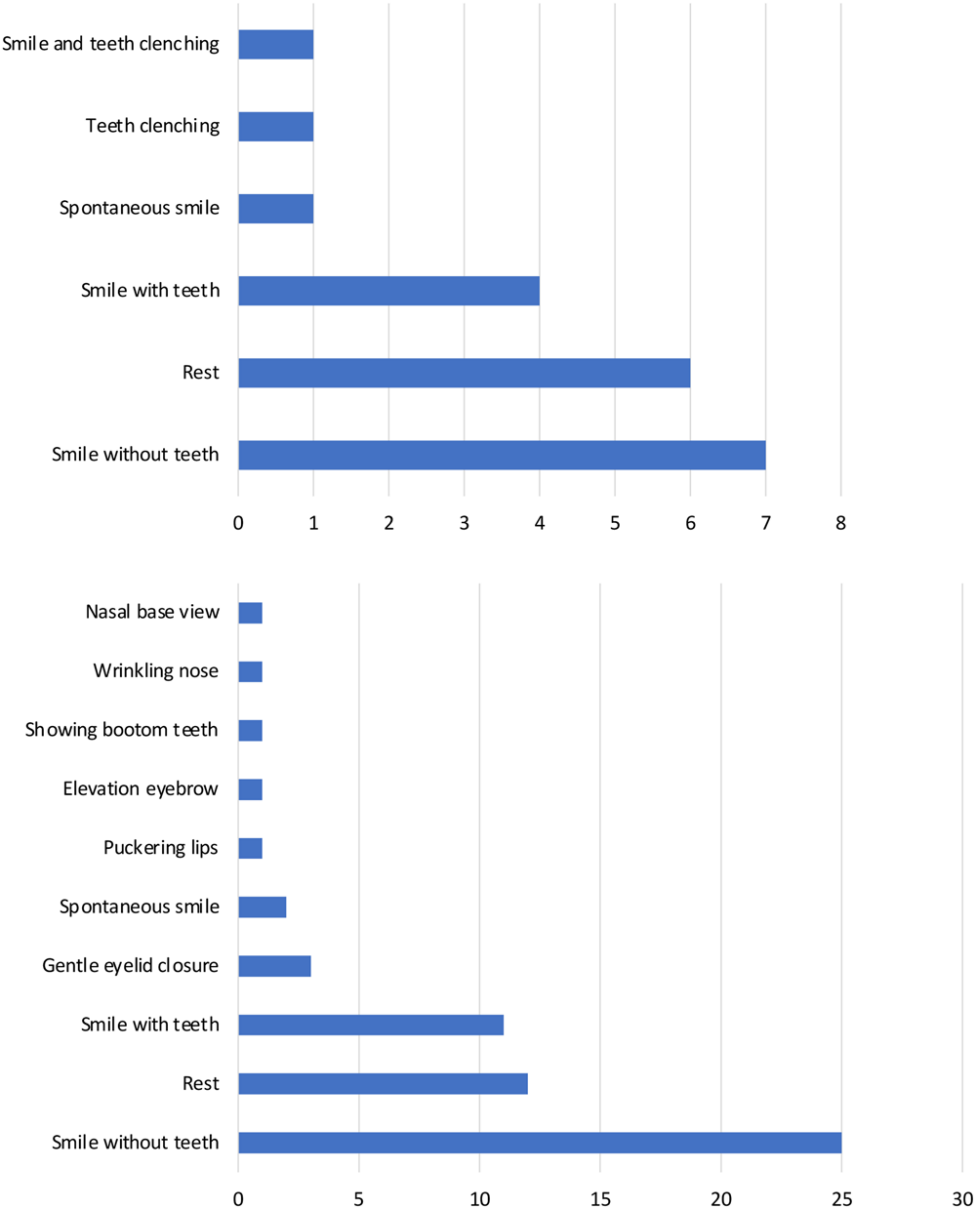
Number of view/emotions used in the landmark recognition software (*above*) and in the emotion recognition software (*below*).

From the included studies, 5 different AI applications for facial assessment were identified. Three of these applications combined symmetry assessment with emotion recognition. The materials required for these assessments were photographs, videos, or both. For emotion recognition, videos were mandatory. The most common photographic views used in symmetry assessments included smile with closed lips (24 studies), neutral/resting face (20 studies), and smile with teeth showing (10 studies). The most common videographic views for emotion analysis were neutral/resting face (4 studies), smile with teeth showing (4 studies), smile with closed lips (3 studies), and spontaneous smile (2 studies) (Fig. 4). Table 1 outlines the characteristics of the identified AI software for facial palsy assessment.

**Table 1.** Specifications of the Identified AI Software for Facial Palsy Assessment

Characteristic	Emotrics	Media Pipe Face Mesh	Real-Time Facial Asymmetry Analysis	FaceReader	Affdex
No. of identified studies	30	1	1	4	2
Software validation study	Yes	No	No	Yes	Yes
Platform	Windows	Windows, Linux, IOS	Windows	Windows	Windows, Linux
Software licensing type	Opensource	Opensource	Proprietary	Proprietary	Proprietary
Land markers	68	468	68	468	6
Action unit intensity	No	No	No	Yes	Yes
Landmark measurement	Brow height, marginal reflex distance, commissure excursion, commissure height deviation, smile angle, upper lip deviation, dental show, lower lip deviation	Depends on programming	Oral displacement, eyebrow displacement	No	No
Emotions recognition	No	No	No	Neutral, happy, sad, angry, surprised, scared, disgusted, contempt, arousal, valence	Neutral, happy, sad, angry, surprised, scared, disgusted, contempt, attention, valence, engagement
Unilateral/bilateral	Both	Depends on programming	Unilateral	Both	No study identified
Tutorial	Yes	No	Yes	Yes	Yes
Data storage	Data stored locally on HD	Depends on programming	Data stored locally on HD	Data stored locally on HD	Data stored locally on HD
User interface	Yes	No	Yes	Yes	Yes
Easy to use	Easy	Difficult	Easy	Medium	Medium
URL	https://github.com/dguari1/Emotrics	https://github.com/google/mediapipe	https://www.h.u-tokyo.ac.jp/plastic/contents/RFAA.html	https://www.noldus.com/facereader	https://www.affectiva.com/emotion-ai/ https://imotions.com/products/imotions-lab/modules/fea-facial-expression-analysis/

HD, hard drive.

## DISCUSSION

Recent exponential increase in volume of publications using AI for facial assessments underscore the role of such technologies in monitoring outcomes after facial reanimation surgery.^24,25^ However, there is a lack of practical guidelines and recommendations for adopting this technology into clinical practice and research protocols. This current systematic review aimed to assess the use of these tools, identify their advantages and limitations, and provide practical guidelines, including a proposed video standard, based on the systematic review and discussion with an interdisciplinary, multicenter expert panel.

### History Objective

The groundbreaking work for objective analysis in the field of facial movement was established in 1999 by Frey et al.^26^ (Table 2), who developed a method for three-dimensional video analysis of facial dynamics using an intricate mirror system. In 2006 and 2008, measurement tools were introduced that used manually placed facial markers, with specialized software for data processing.^27,28^ In 2012, Facegram was introduced and widely adopted in numerous studies related to facial reanimation surgery.^29^ However, it still requires users to manually identify facial landmarks.

**Table 2.** History of Selected Publications Using Facial Assessment Tools for Facial Palsy Patients

Year	Author	Country	Assessment Method
1999	Frey	Austria, Switzerland	Three-dimensional video analysis of facial movements
2005	Manktelow	Canada	Facial reanimation measurement system
2008	Aubà	Spain	FACIAL CLIMA
2012	Hadlock	United States	Facegram
2018	Guarin	England	Emotrics
2019	Dusseldorp	Australia	Affdex
2020	Boonipat	United States	FaceReader

By the mid to late 2010s, significant advancements in facial landmark recognition technology were achieved, because of deeper and more sophisticated neural networks. These improvements greatly enhanced the accuracy and efficiency of facial analysis software. In 2018, Emotrics was introduced.^30^ Our study highlights Emotrics as the first AI-driven tool for assessing outcomes of facial reanimation surgery, marking a significant milestone in the field.

**Fig. 5.**
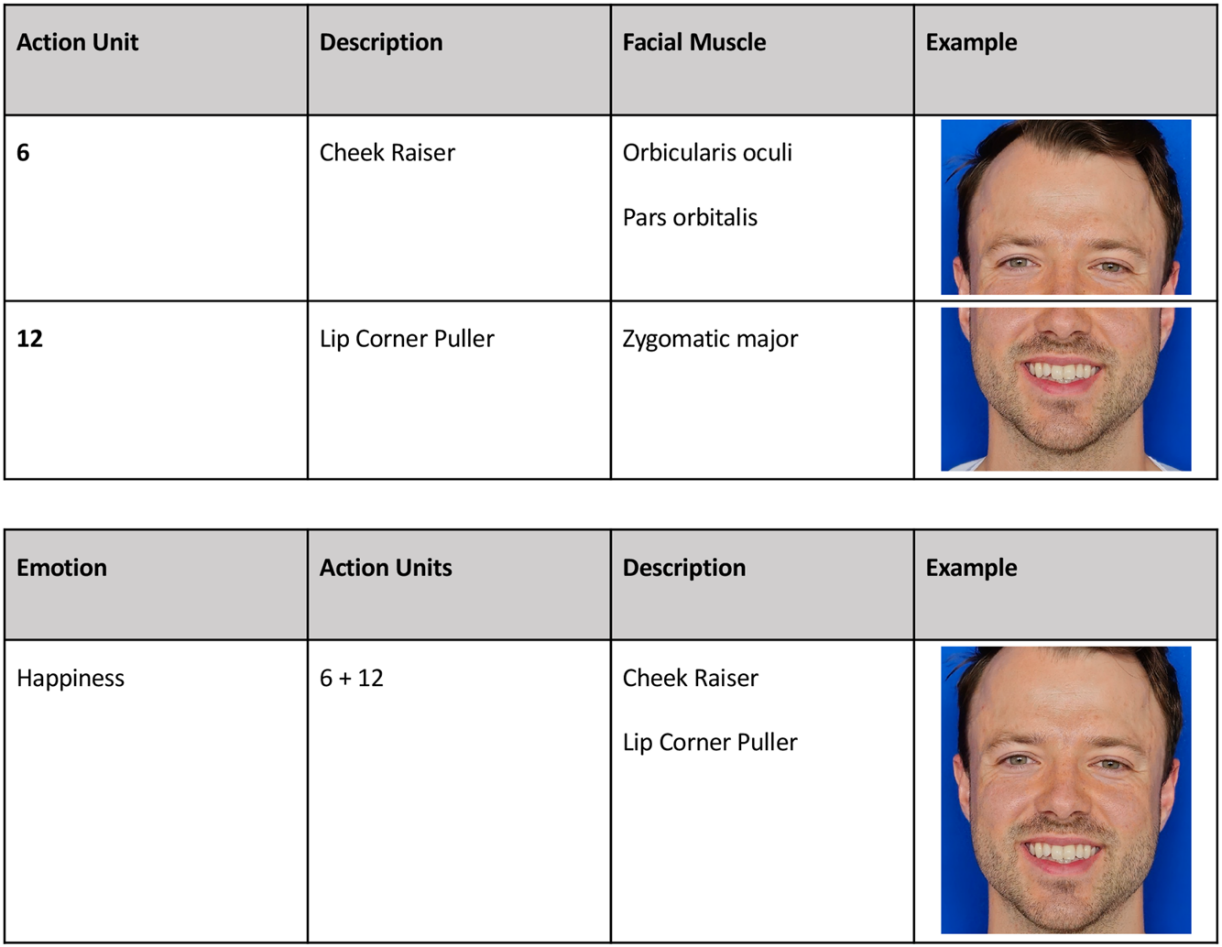
Example of FACS. Emotions are identified on the collective activation of the AUs. For example, happiness is calculated from the combination of AUs 6 (cheek raiser) and 12 (lip corner puller).

The most recent advancements in the field include facial expression engines such as FaceReader and Affdex. They are equipped to analyze facial expressions using the Facial Action Coding System (FACS), a comprehensive and standardized system for classifying facial expressions. Within this software, the FACS is operationalized through action units (AUs), where each AU corresponds to specific facial muscles or groups of muscles. The emotion is determined by the combination of various AUs^31^ (Fig. 5).

### Significance of the AI Programs in Facial Palsy

Currently, our expert panel sees that facial palsy outcomes are being evaluated across 5 key levels. These include clinician-guided grading systems, patient-reported outcome measures, quantitative photographic tools, layperson assessments, and spontaneity assessments. Within these categories, AI-powered tools have demonstrated significant potential. For instance, Emotrics has proven to be an efficient quantitative photographic tool,^32^ whereas emotion detection programs, such as FaceReader and Affdex, facilitate layperson assessments.^33^ These AI tools are particularly valuable, as they help mitigate the resource-intensive demands of traditional methods, both in terms of personnel and data privacy concerns. As such, they have the potential to complement existing outcome assessment tools and, in some cases, replace traditional severity grading methods.^34^ Moreover, there is growing research on AI applications for spontaneity assessment and the automation of clinician-led grading systems, such as Electronic Facial Paralysis Assessment. However, a notable limitation remains the accurate quantification of synkinesis, which continues to present challenges, even for advanced AI software.

### Scope of AI-Software Applications

The systematic review revealed that a broad spectrum of facial palsy causes, clinical manifestations, and a diverse array of interventions and operations for treating facial palsy are suitable. These include unilateral and bilateral palsy cases stemming from different causes, affecting individuals ranging from 3-year-old children^35^ to the older population up to 90 years.^36,37^ Moreover, there were no reported issues regarding compliance with children. Using videos to demonstrate and assess emotions proved to be an effective method. In the context of static procedures, AI demonstrates capability in assessing symmetry and emotion recognition in a wide range of therapies, such as neurotomies, placement of eyelid weights, tensor fascia lata slings, muscle resections, and lateral tarsal strips.^22,36,38–42^ Regarding dynamic operations, AI can assist in the objective evaluation of local and free functional muscle transfers, and nerve transfer. These can be innervated singularly or dually through cross-facial nerve grafts, the masseter, hypoglossus, or accessory nerves.^17–20,23,32–35,37,43–58^ Furthermore, it encompasses interventions such as botulinum toxin injections.^59,60^

### AI Software Selection

**Fig. 6.**
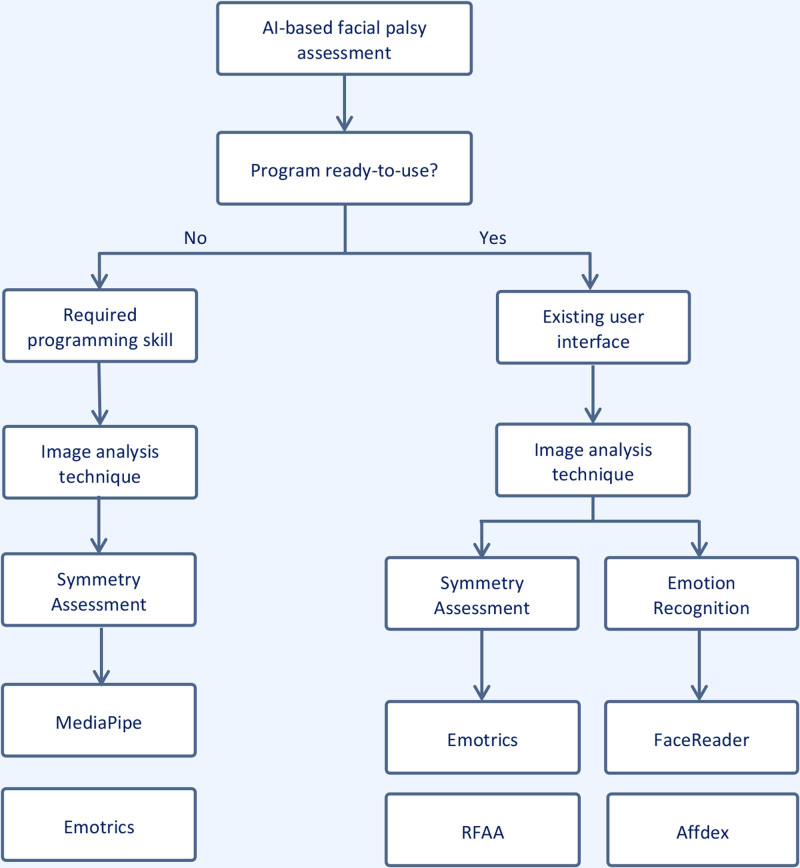
This flowchart provides a step-by-step guide for clinicians to select the appropriate AI-based facial assessment software. Combining landmark comparison and emotion recognition software may be necessary to address the research hypothesis. *RFAA*, real-time facial asymmetry analysis.

In the rapidly evolving field of facial palsy treatment and assessment, choosing the appropriate facial assessment application can be challenging. To aid in the software decision-making process, the proposed flowchart categorizes software based on specific clinical needs and technical capabilities (Fig. 6).

**Fig. 7.**
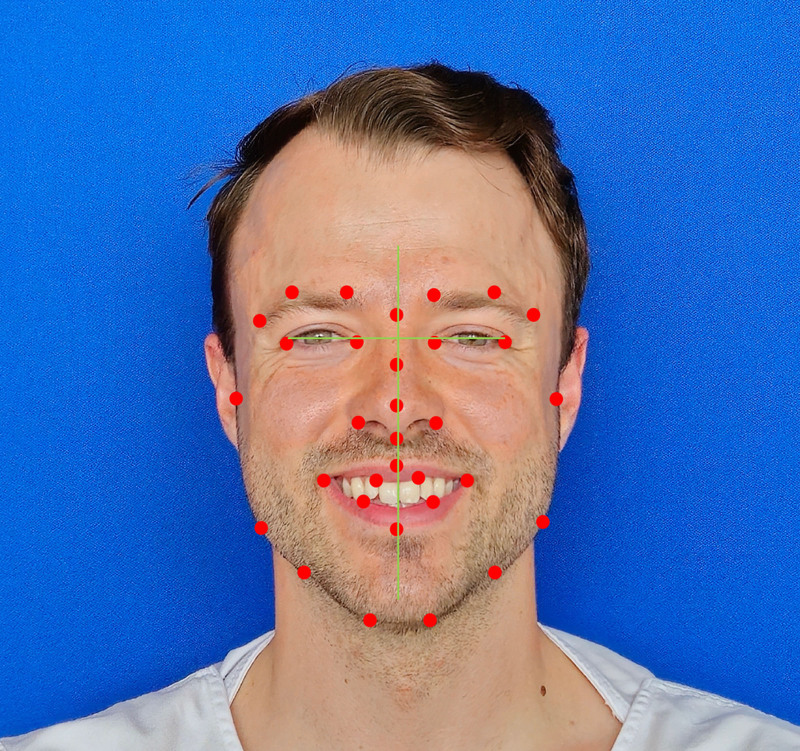
AI symmetry measurement of a healthy patient. Exemplary photograph, showing the automatically detected facial landmarks (*red dots*) and facial axis (*green*). The software then calculates the symmetry of different structures (eg, brow, smile angle).

Emotrics assesses symmetry by automating the placement of facial landmarks on images to produce various measurements. Its use was first highlighted by Guarin et al. in 2018 and further enhanced with a dedicated facial palsy database in 2020 (Fig. 7). As the most commonly used AI tool in this systematic review, featured in 30 studies, Emotrics is praised for its user-friendly interface and free access. However, it is limited to analyzing frontal images and specific measurements such as brow height, marginal reflex distances, commissure excursion, and smile angle, among others. It lacks capabilities for real-time assessment and emotion recognition (https://www.sircharlesbell.com/).

**Fig. 8.**
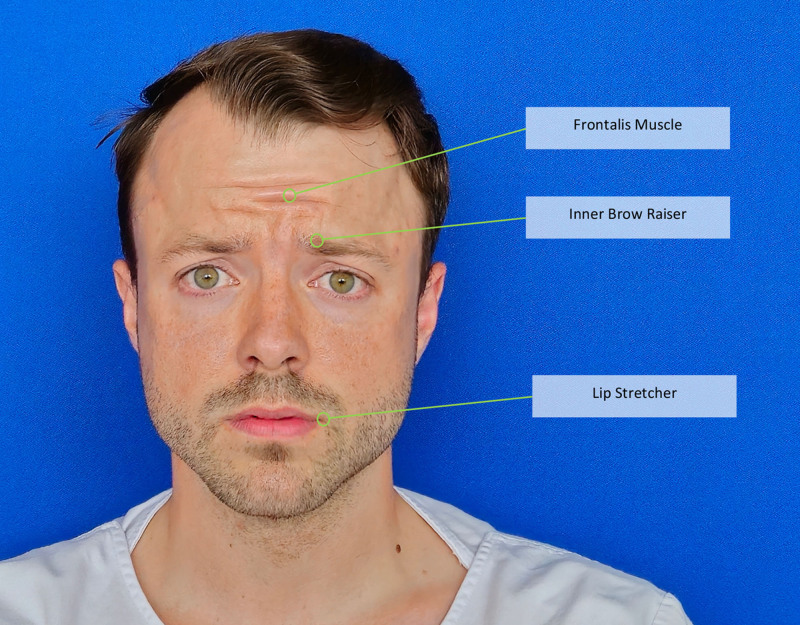
AI emotion recognition. Exemplary graphic user interface. Set of facial muscle movements that correspond to the displayed emotion (fear).

FaceReader (Noldus Information Technology, Wageningen, The Netherlands), has made substantial contributions to facial expression analysis (Fig. 8), with over 35,000 citations in scientific publications by 2022. Capable of discerning emotions from frontal and slightly tilted faces, it has been used in 4 studies within our systematic review. Although proprietary and requiring purchase, Noldus offers dedicated support and customization for research-specific needs (https://www.noldus.com/contact).

Affdex (Affectiva, Boston, MA), mentioned in a single study from our review, is designed to identify 7 basic emotions (anger, disgust, fear, joy, sadness, surprise, and contempt) plus neutrality, using predictions from 20 AUs. This software demands programming expertise for integration into applications. The company has shifted focus toward market and media research, social robotics, and behavioral studies. For facial palsy research, it now promotes its sister company, iMotions, which includes facial expression analysis as a module. iMotions comes at a slightly higher price point than FaceReader (https://imotions.com/de/).

MediaPipe Face Mesh (Google LLC Mountain View, CA) was used in 1 study to identify three-dimensional facial landmarks from two-dimensional clinical photographs of patients. Although the model is available for free download, it requires programming expertise and lacks a user-friendly interface (https://github.com/google/mediapipe).

The Real-Time Facial Asymmetry Analysis is used in one study, using AI to evaluate facial asymmetry with a focus on the oral and eye regions. It offers real-time computation, works on a standard laptop, and is user-friendly. It is freely available and easy to install as an executable file and does not require programming skills (https://www.h.u-tokyo.ac.jp/plastic/contents/RFAA.html).

### Videographic Standard

Although photographic standards are well established in the facial palsy field,^61^ no written consensus of universal videographic standards for emotion has yet been developed. Our literature search revealed studies using a wide range of videographic views and emotional expressions in various AI applications. However, although photographic standards are well established, not all views were mentioned or analyzed in the identified studies. Most often, symmetry was assessed from only 1 or 2 views, likely because of feasibility considerations. We believe that including a complete set of preoperative and postoperative views would be feasible and could be easily demonstrated in a table. Most commonly, rest and different smiles were analyzed, likely because of the prevalence of dynamic smile reanimation procedures. For emotion recognition, videographic views of patients’ resting position and their various smiles, indicating happiness, were assessed. However, the identified AI applications, such as FaceReader and Affdex, are capable of recognizing a broader range of emotions. They used slightly different names and sets of emotions but consistently identified the 7 basic emotions based on universal facial expressions, as outlined in the FACS by Ekman and Rosenberg.^62^

**Fig. 9.**
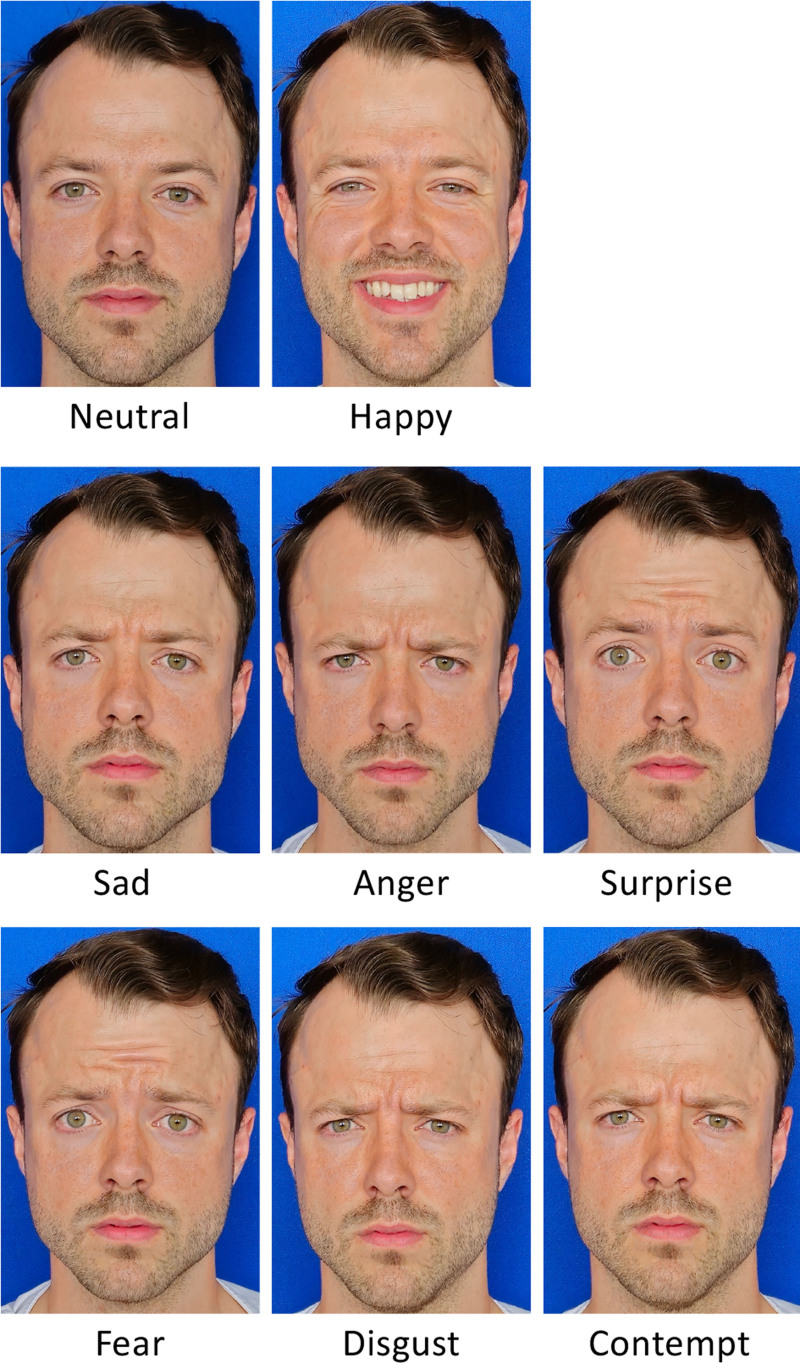
Proposed standardized videographic set that should be used in outcome analysis to improve data comparison and information exchange.

Therefore, we recommend using the photographic views proposed by Santosa et al.^61^ for videographic assessments and focusing on the validated emotions: neutral and happy. Although other emotional expressions may be of interest, further validation studies are necessary before they can be adopted as standards. For instance, Dusseldorp et al. previously attempted to establish an emotionality quotient, incorporating negative emotions such as disgust and contempt.^23^ Similarly, Kollar et al. identified significant changes in emotions such as sadness and disgust.^34^ With further validation, the inclusion of emotions such as sadness, anger, surprise, fear, disgust, and contempt (Fig. 9) could become valuable. These emotions have already been integrated into other fields, such as face transplantation.^63^ It is important to note that we suggest evoking the emotions voluntarily. Dusseldorp et al. use the concept of the spontaneous emotions.^19^ A spontaneous smile, elicited unconsciously, is more natural but requires a time-intensive setup. Both smile types have been validated in facial palsy studies, with voluntary expressions proving effective in outcome measurements, especially as tools such as FaceReader were trained on databases featuring voluntary emotions.^17,19,23,34,37,64^

### Data Safety

Since the software (Emotrics, RFA, FaceReader, Affdex) is installed directly onto desktop computers, data security is considerably enhanced. This local storage solution ensures that all sensitive data are stored on the hard drive of the user’s computer. Such a configuration mitigates risks associated with data breaches and unauthorized external access.

### Limitations

Our review has several limitations. The focus solely on AI-based assessment tools might limit the scope of our review, potentially overlooking significant contributions from non–AI-based or hybrid assessment methods. In addition, the exclusion of editorials, letters, narrative reviews, case reports, conference abstracts, and trial registry entries might restrict our discussion on the evolving landscape and emerging challenges within the field. Even though all AI tools were validated, only 5 studies were validation studies.^17,19,23,34^ All studies were retrospective and have a moderate risk of bias. This underscores the need for more high-quality studies with robust validation.

## CONCLUSIONS

The evolution of AI tools in facial reanimation surgery offers promising and efficient advancements for objective assessment. The tools work across different patient groups and various facial palsy treatment strategies. The proposed video standard for emotion analysis may help to design future validation and prospective studies.

## DISCLOSURE

The authors have no financial interests to declare in relation to the content of this article.

## ACKNOWLEDGMENTS

This research was supported by a research grant by the Gottfried and Julia Bangerter-Rhyner Stiftung. The authors thank Lorenz Klaus for serving as the model for their photographic and video materials. A machine learning natural language processing model was used for grammatical and spelling corrections. The authors are fully responsible for the originality, validity, and integrity of the content of their article and have ensured that it complies with publication ethics policies.

## PHOTOGRAPHIC CONSENT

The subject provided written informed consent for the use of his images.

## Supplementary Material
