# Supplementary material for: Artificial Intelligence in Facial Palsy Treatment: A Systematic Review and Recommendations
Source: Plast Reconstr Surg. 2025 Mar 18;156(3):477–90. doi: 10.1097/PRS.0000000000012105 (PMC12376819; doi:10.1097/PRS.0000000000012105)
Supplement: Supplementary file 1 [file prs-156-0477-s001.pdf]

Figure, Supplemental Digital Content 1. Detailed search strategy for the systematic review, including database queries and the study selection process.

S. M. systematically searched Medline, Embase and the Cochrane Central Register of Controlled Trials (CENTRAL) using text words and database-specific subject headings (last date of search: February 2nd, 2024). The search strategy was initially drafted for PubMed, translated with the Polyglot Search Translator to the other databases and peer-reviewed by P.P. All retrieved references were exported to Covidence and duplicates were removed. Two reviewers (SM, PP) independently screened all references resulting from the search. They then independently screened all potentially eligible articles in full-text for eligibility. Disagreements were solved by consensus/by a third reviewer.

PubMed Medline

Search date 18.02.2024; PubMed ALL to February 18, 2024, 708 hits

(("Facial Paralysis"[MeSH] OR "Bell Palsy"[MeSH])

OR

((face[tw] OR faces[tw] OR facial[tw] OR visage[tw] OR orofac*[tw] OR "oro-facial"[tw] OR temporal[tw] OR zygomatic [tw] OR buccal [tw] OR mandibular[tw] OR cervical [tw] OR eyelid[tw] OR mouth OR lip[tw] OR lips[tw] OR smile[tw] OR forehead[tw] OR platysma[tw])

ADJ3

(neuroparalysis[tw] OR paralys*[tw] OR palsy[tw] OR paresis[tw] OR pals*[tw] OR parlayz*[tw] OR asymmetr* OR impair* OR reanimat*[tw]))

OR

("nerve injury"[tw] OR "bell palsy"[tw] OR "bells palsy"[tw] OR "Ramsay-Hunt syndrome"[tw] OR moebius[tw] OR “moebius syndrome” OR "nerve damage"[tw] OR monoplegia[tw] OR "peripheral nerve injury"[tw]))

OR

(("Artificial Intelligence"[MeSH] OR "Software"[MeSH] OR "Machine Learning"[MeSH])

OR

(“artificial intelligence”[tw] OR software[tw] OR “machine learning”[tw] OR "AI"[tw] OR "artificial neural networks" [tw] OR "cognitive computing"[tw] OR "autonomous systems"[tw] OR “expression analysis”[tw] OR “assessment tool”[tw] OR "machine learning"[tw] OR "deep learning"[tw] OR "neural networks"[tw] OR "pattern recognition"[tw] OR "image recognition"[tw] OR "natural language processing"[tw] OR stereophotogrammetry[tw] OR "computer vision"[tw] OR "predictive analytics"[tw] OR "intelligent systems"[tw] OR "data mining"[tw] OR “root mean square”[tw] OR “open source”[tw] OR “open-source”[tw] OR “java”[tw] OR facegram[tw] OR emotrics[tw] OR faap[tw] OR facogram[tw] OR “auto eface” [tw] OR “auto-eface” [tw] OR sbface[tw] OR affdex[tw] or facereader[tw]))

NOT (animals/NOT humans/)
