# Supplementary material for: Artificial Intelligence in Facial Palsy Treatment: A Systematic Review and Recommendations
Source: Plast Reconstr Surg. 2025 Mar 18;156(3):477–90. doi: 10.1097/PRS.0000000000012105 (PMC12376819; doi:10.1097/PRS.0000000000012105)
Supplement: Supplementary file 2 [file prs-156-0477-s002.pdf]

Table, Supplemental Digital Content 2. A list of the 35 studies included in the systematic review, highlighting their key characteristics and relevance to the analysis.

Author	Year	N	Study Type	AI-Tool	Validation	ROBINS-I Risk of Bias	Type of Procedure	Follow-up (m)
Bianchi[31]	2022	8	Retrospective	Emotrics	No	Moderate	Dynamic	22.8
Boonipat[17]	2020	15	Retrospective	FaceReader	Yes, comparison of joy, negative emotion and neutral signal comparing patients and healthy subjects.	Moderate	Dynamic	
Cabañas Weisz[34]	2021	41	Retrospective	Emotrics	No	Moderate	Static	38.2
Derakshan[36]	2022	43	Retrospective	Emotrics	No	Moderate	Static	6.8
Dusseldorp[19]	2022	39	Retrospective	Emotrics, Affdex	Yes, comparison of joy and negative emotion comparing patients and healthy subjects.	Moderate	Dynamic	
Dusserdorp[20]	2019	49	Retrospective	Emotrics	No	Moderate	Dynamic	9.6
Dusserdorp[23]	2019	134	Retrospective	Affdex	Yes, emotionality index was corrrelated with layperson assessment. Healthy subjects were analyzed as a comparison.	Moderate	Static, Dynamic	
Ein[35]	2023	20	Retrospective	Emotrics	Yes, Emotrics-derived nasolabial fold data was matched with corresponding eFACE parameters.		Dynamic	31
Ferri[57]	2023	9	Retrospective	Emotrics	No	Moderate	Chemodenervation	
Gray[41]	2020	8	Retrospective	Emotrics	No	Moderate	Dynamic	
Greene[33]	2022	113	Retrospective	Emotrics	No	Moderate	Dynamic	30.7
Greene[22]	2019	53	Retrospective	Emotrics	No	Moderate	Static	25
Hidaka[42]	2023	23	Retrospective	Individual	No	Moderate	Dynamic	24.3
Hohman[43]	2023	5	Retrospective	Emotrics	No	Moderate	Dynamic	20.6
Kaufmann Goldberg[37]	2024	56	Retrospective	Emotrics	No	Moderate	Static	19.5
Kaufman Goldberg[45]	2023	13	Retrospective	Emotrics	No	Moderate	Dynamic	16.2
Kaufmann Goldberg[44]	2023	55	Retrospective	Emotrics	No	Moderate	Dynamic	19.5
Kaufmann Goldberg[38]	2023	55	Retrospective	Emotrics	Correlation between smile excursion, interlabial distance asymmetry and subjective favorable outcome was ranked	Moderate	Static	8
Kollar[32]	2023	12	Retrospective	Emotrics, FaceReader	No	Moderate	Static, Dynamic	40
Kollar[18]	2023	20	Retrospective	Emotrics, FaceReader	No	Moderate	Dynamic	31.8

Kollar[46]	2022	63	Retrospective	FaceReader	Yes, positive correlation of Terzis Score with the IS of happiness emotion	Moderate	Dynamic	14.7
Krag[58]	2021	36	Retrospective	Emotrics	No	Moderate	Chemodenervation	
Krane[30]	2021	20	Retrospective	Emotrics	No	Low	Dynamic	14.7
Lee[47]	2023	59	Retrospective	Emotrics	No	Moderate	Dynamic	34
Mabvuure[49]	2022	10	Retrospective	Emotrics	No	Moderate	Dynamic	39.2
Mabvuure[48]	2020	41	Retrospective	Emotrics	No	Moderate	Dynamic	26.4
Miller[39]	2021	19	Retrospective	Emotrics	No	Moderate	Static	
Park[50]	2022	21	Retrospective	Emotrics	No	Moderate	Dynamic	
Sanchez[40]	2023	79	Retrospective	Emotrics	No	Moderate	Static	22
Thachil[51]	2023	47	Retrospective	Emotrics	No	Moderate	Dynamic	72
Tzafetta[52]	2020	11	Retrospective	Emotrics	No	Moderate	Dynamic	48
Valencia-Sanchez[53]	2023	11	Retrospective	Emotrics	No	Moderate	Dynamic	30.4
Weiss[55]	2023	46	Retrospective	Emotrics	No	Moderate	Dynamic	24.9
Weiss[54]	2022	28	Retrospective	Emotrics	No	Moderate	Dynamic	27.6
Woo[56]	2023	35	Retrospective	Individual	No	Moderate	Dynamic	12
